# DR*W201/P65 Tetramer Visualization of Epitope-Specific CD4 T-Cell during *M. tuberculosis* Infection and Its Resting Memory Pool after BCG Vaccination

**DOI:** 10.1371/journal.pone.0006905

**Published:** 2009-09-04

**Authors:** Huiyong Wei, Richard Wang, Zhuqing Yuan, Crystal Y. Chen, Dan Huang, Lisa Halliday, Weihua Zhong, Gucheng Zeng, Yun Shen, Ling Shen, Yunqi Wang, Zheng W. Chen

**Affiliations:** 1 Department of Immunology & Microbiology, Center for Primate Biomedical Research, University of Illinois College of Medicine at Chicago (UIC), Chicago, Illinois, United States of America; 2 Biological Resource Laboratory, University of Illinois at Chicago (UIC), Chicago, Illinois, United States of America; McGill University, Canada

## Abstract

**Background:**

In vivo kinetics and frequencies of epitope-specific CD4 T cells in lymphoid compartments during *M. tuberculosis* infection and their resting memory pool after BCG vaccination remain unknown.

**Methodology/Findings:**

Macaque DR*W201 tetramer loaded with Ag85B peptide 65 was developed to directly measure epitope-specific CD4 T cells in blood and tissues form macaques after *M. tuberculosis* infection or BCG vaccination via direct staining and tetramer-enriched approach. The tetramer-based enrichment approach showed that P65 epitope-specific CD4 T cells emerged at mean frequencies of ∼500 and ∼4500 per 10^7^ PBL at days 28 and 42, respectively, and at day 63 increased further to ∼22,000/10^7^ PBL after *M. tuberculosis* infection. Direct tetramer staining showed that the tetramer-bound P65-specific T cells constituted about 0.2–0.3% of CD4 T cells in PBL, lymph nodes, spleens, and lungs at day 63 post-infection. 10-fold expansion of these tetramer-bound epitope-specific CD4 T cells was seen after the P65 peptide stimulation of PBL and tissue lymphocytes. The tetramer-based enrichment approach detected BCG-elicited resting memory P65-specific CD4 T cells at a mean frequency of 2,700 per 10^7^ PBL.

**Significance:**

Our work represents the first elucidation of in vivo kinetics and frequencies for tetramer-bound epitope-specific CD4 T cells in the blood, lymphoid tissues and lungs over times after *M. tuberculosis* infection, and BCG immunization.

## Introduction

CD4 T cells play a critical role in immune protection. CD4 T cells function through their capacity to help B cells produce antibodies, to activate macrophages for enhanced microbicidal activity, to recruit leukocytes to infection/inflammation sites, and through their production of cytokines and chemokines to orchestrate adaptive immune responses. Naïve conventional CD4 T cells can evolve to at least four distinct cell populations, Th1, Th2, Th17 and induced regulatory T cells (iTreg), in response to antigens. Since signaling triggered by class II MHC/peptide-TCR interaction and cytokine environment are important for activation and differentiation of naïve CD4 T cells, the evolution of these four T cell populations after antigen exposure or infections may depend upon the pattern of signals they receive during the initial interaction with antigen [1]. Direct tracking of class II MHC-restricted epitope-specific CD4 T cells will help to elucidate evolution or differentiation of Th1, Th2, Th17 and Treg cell populations in development of balanced anti-microbial immunity against viral or bacterial infections.

*Mycobacterium tuberculosis*-induced tuberculosis remains to be a major killer among infectious diseases. Studies in mice and HIV-infected humans indicate that CD4 T cells are of central importance for immune protection against tuberculosis [2], [3]. While Th1 cytokines IFN-γ and TNF-α are critical for immune protection against tuberculosis in mice, the role of human TNFα anti-tuber culosis immunity is demonstrated in the subjects who receive anti-TNF mAb treatment of rheumatoid arthritis, and develop reactivation tuberculosis [4], [5]. Given the possibility that human Th1, Th2, Th17 and Treg cells play a role in immune regulation of tuberculosis, it is important to determine the evolution and interrelation of these CD4 T cell populations in primary *M. tuberculosis* infection. Developing better assay systems for direct measurement of MHC-restricted epitope-specific CD4 T cells should be an important step toward in-depth studies of evolution and immune function of Th1, Th2, Th17 and Treg during primary *M. tuberculosis* infection.

The recent development of tetrameric or multimeric MHC/peptide complexes with the capacity to bind TCR has provided a useful tool to study antigen-specific T cell responses directly *ex vivo* [6], [7], [8], [9], [10]. Class I MHC/peptide tetramers have revolutionized the identification, enumeration, and phenotyping of antigen-specific CD8^+^ T cells [8], [11], [12], [13], but the potential of class II MHC/peptide tetramers has not been adequately appreciated due to technical problems for generating useful class II MHC/peptide tetramer and for measuring low-frequency Ag-specific CD4^+^ T helper cells. Despite the fact that class II MHC tetramers loaded with autoantigen peptides have been well-documented [see review in reference [10]], only limited numbers of class II MHC/microbial peptide tetramers were developed to measure microbial epitope-specific CD4 T cells [9], [14], [15], [16], [17]. Although class II MHC tetramer-based analysis may open a new avenue to further understand differentiation and evolution of antigen-specific CD4 T-cell subpopulations in the context of vaccinations or infections, fundamental aspects of the tetramer application remain to be characterized. While tetramer-based assay is presumably advantageous over peptide-stimulation-based intracellular cytokine staining assay in terms of direct/rapid measurement, objective enumeration, and simultaneous phenotyping analyses, comparative evaluation of these two assays during mycobacterial infection and vaccination has not been done. Importantly, the utility of MHC/peptide tetramers in documenting fine development of epitope-specific CD4 T cell responses in the blood, lymphoid tissues and infected lungs during primary *M. tuberculosis* infection has not been determined [18]. The paucity of the information is somehow attributed to the fact that murine class II MHC tetramers for mycobacterial antigens are not available for application in mouse models of tuberculosis. Furthermore, the possibility that the class II MHC tetramer is able to confer direct and rapid measurement of *Mycobacterium Bovis* Bacillus Calmette-Guérin (BCG) vaccine-elicited resting memory CD4 T cells remains largely unknown. To address these fundamental questions, we have generated macaque class II MHC/Ag85B peptide tetramers. We then evaluated standard tetramer direct staining, tetramer staining after peptide stimulation, and tetramer-based enrichment approaches for direct measurements of epitope-specific CD4 T cells in blood, lungs, lymph nodes and spleens during *M. tuberculosis* infection of Mamu-DRB*W201^+^ rhesus macaques. We also employed the tetramer-based enrichment approach to successfully measure BCG-elicited resting memory P65-specific CD4 T cells 2 years after vaccination. Our studies represented the first elucidation of kinetics for tetramer-bound epitope-specific CD4 T cells in animal models of tuberculosis/vaccination.

## Materials and Methods

### Ethic Statement

The use of macaques and experimental procedures were approved by Institutional Animal Care and Use Committee and Biosafety Committee, University of Illinois College of Medicine at Chicago (UIC), and we followed the national and international guidelines regarding "The use of non-human primates in research to minimize potential suffering of the studied macaques. Daily or weekly clinical follow-up were taken to ensure that animals were not suffering from severe coughing, respiratory distress, depression, refusion to take food, body-weight loss or other potential life-threatening signs. Human euthanization procedures were immediately taken if those signs occur progressively.

### Proliferation assays to *Mycobacterium* antigen Ag85B peptides in BCG vaccinated macaques

Twelve Indian rhesus macaques (*Macaca mulatta*) were vaccinated intravenously with 10^6^ CFU of BCG and used screening epitope peptides of Ag85B. Conventional proliferation assay was carried out as described previously [19]. Briefly, Sixty nine 15mer peptides overlapping by 11mer and spanning entire Ag85B protein (synthesized by Genscript) were divided in 10 groups, 7 peptides per group (6 for group 10). Macaque PBL (1×10^5^ cells per well) were cultured in triplicate of 96-well plates in presence of one of ten groups of Ag85B peptide pools, individual 15mer peptides, purified protein derivative of tuberculin (PPD)(1, 5 or 25 µg/ml), ConA (5 µg/ml), bovine serum albumin (BSA 3 µg/ml), or medium alone. Five days later, cells were pulsed with ^3^H-thymidine at 1.0 uCi per well, and uptake was measured 8 hours later using a 1450 Mirocbeta scintillation counter (Wallac, Gaithersburg, MD). Stimulation index was defined as the ratio of the mean CPM of PPD-, peptide group- or ConA-stimulated wells relative to the mean CPM of control wells (medium alone).

### Determination of Mamu-DR alleles

To examine the associated class II MHC molecules in these BCG-vaccinated macaques and identify Mamu-DR alleles in naïve monkeys for further immune studies, *Mamu-DRB* phenotype of each macaque was screened using nest-PCR and DNA sequence analysis. All macaques were collected 0.5 ml of anti-coagulated blood to extract genomic DNA using DNeasy Blood & Tissue Kit (Qiagen), the genomic DNA samples were used to amplify the full-length *Mamu-DRB* fragment by a standard PCR with a forward primer (5′-GCCCGTCGACCTGTCCTGTTCTCCAGCATG-3′) and a reverse primer (5′-GGCGGGATCCCTTTTCATCCTGCAAAGCTG-3′) as previous described [20], as the α-chain is usually identical in all Mamu class II variants. Subsequently, a pair of primers (5′-CCGCTCCAGGATGTCCTCCC/5′-CTCGAGTGTCCCCCCAGCACGTTTC) were used in a second-round PCR to amplify the exon 2 of β1 domain in DRB*W201 chain [15]. The separated DNA fragments with the exact predicted sizes on the agarose gel were collected and purified by QIAquick Gel Extraction Kit (Qiagen) and then directly sequenced. The *Mamu-DRB* allele of individual macaques was determined by sequence analysis using the Genbank data base [15], [20].

### Pulmonary *M. tuberculosis* infection in macaques

Six Chinese rhesus macaques, three DRB*W201^+^ and three DRB*W201^-^ were infected with *Mycobacterium tuberculosis* to measure kinetics of epitope-specific CD4 T cells in different lymphoid compartments. 500 CFU of *M. tuberculosis* Erdman (validated stock from FDA) in 2 ml PBS were administered into the right caudal lung lobe of each macaque using bronchoscope (Olympics) in BRL Annex BSL3 monkey facility, as we recently described [21]. Blood samples were collected from individual animals after vaccination or infection to isolate PBL for evaluating T-cell responses. Lung, axillary and periaortic lymph nodes, and spleens were also collected at necropsy to isolate lymphocytes for tetramer staining.

### Construction of MHC class II plasmids with Ag85B P65 covalent loading

PBL collected from macaques were used to extract RNA using the TRIzol-based (Invitrogen) isolation method and the cDNA was synthesized using the First Strand cDNA Synthesis Kit (Clontech Lab). Two pairs of oligonucleotide primers were synthesized (Operon) to amplify the full-length DNA fragments of *Mamu-DR*α and β chain by RT-PCR respectively. The PCR products were cloned into vector *pCR2.1* using TA cloning kit (Invitrogen) and then determined their nucleotide sequences.

To generate the soluble *Mamu-DR* molecules, PCR-based recombination technique was used to construct chimeric genes encoding the extracellular DR α-Jun and epitope-extracellular DR β-Fos-BSP chains as we and others previous described [6], [7], [9], [12], [22]. Briefly, Mamu DRα *0101 cDNA sequences encoding extracellular domain was recombined with Jun-coding sequence and then subcloned into the inducible vector *pMT/V5-His A* (Invitrogen). The sequence encoding the *Mycobacterium* Ag85B-P65 (258∼272aa, PNGTHSWEYWGAQLN) was fused to the N terminus of the Mamu-DRB* W201 extracellular domains via a flexible polyglycine linker, and then linked to the Fos domain. BirA biotinlyation sequence then added to the P65-DRβ-Fos fragment. The complete epitope-DRβ-Fos-BSP cassette was subcloned into the expression vector *pMT/V5-His A*. As a control, a DNA construct encoding soluble DR306 β chain covalently loaded with a Hsp65 epitope P1 [23] and fused with the Fos and Bir A was similarly developed.

### Selection of transfected cell clones capable of secreting Mamu-DR αβ monomer

*Mamu-DR*/P65 molecules were expressed in *Drosophila* Schneider 2 cells using *Drosophila* Expression System Kit (Invitrogen) as we recently described [22]. Briefly, the equal amount of DRα-Jun and P65-DRβ-Fos-BSP constructs, together with the plasmid *pCoBlast* which carries the *Blasticidin* resistance marker, were co-transfected S2 cells via a standard calcium phosphate transfection technique. The post-transfected cells were selected with *Blasticidin* (Invitrogen) at 25 µg/mL for 3∼4 weeks to establish stable cell colonies as our previous described. The expanded cells were extracted DNA samples to identify the integrated DRα or β-chain using a pair of specific primers as following: 1. Forward primer 5′-AAGCGCTCCAACAACACTCCAATC and reverse primer 5′-TTCTGCGCTTTCAAGGTTTTCACT were synthesized to amplify DRα* 101 chain. 2. Forward primer 5′-AGCCCCTGCAGCACCACACC and reverse primer 5′-GTCGTTCAGTCCACCGCCACCTC were used to amplify DRβ* W201 chain. These α^+^ β^+^ cell lines were induced the production of soluble *Mamu-DR* molecules by adding 500 µM CuSO_4_ for 3 days, and then collected the supernatants by centrifugation. Then the concentrated supernatants were performed dot-blot assay to identify whether recombinant *Mamu-DR* αβ chains have been expressed. Subsequently, these polyclonal cell lines that confirmed expression of *Mamu-DR* molecules were performed two rounds of limited dilution assays to obtain a stable monoclonal cell line that co-expresses DRα and DRβ chains simultaneously as our previous described. The established individual clones were identified by PCR and dot-blot assays again.

### Identification of recombinant *Mamu-DR* molecules by dot-blot and PAGE analysis

The conformational antibody that reacted only with native MHC class II αβ molecules expressed in the surface of APC was used to determine whether recombinant *Mamu-DR* molecules mimic its native configuration that we desired. A prepared PVDF membrane (0.45µ, Millipore) were loaded to different samples from the concentrated supernatants or the purified solutions, and then blocked by PBS blocking buffer (Pierce) for 1 hour with gentle agitation. After washing four times with 0.05% (v/v) Tween-20 in PBS (PBST), the membrane was incubated with 1∶1000 dilution of anti-HLA-DR antibody (clone L243, BD Bioscience, cross-reaction with *Mamu-DR* molecules) for 1 hour. The washed membrane was incubated with 1∶5000 dilution of goat anti-mouse HRP conjugated Ab (Bio-Rad) for 1 hour, and developed using Supersignal West Pico Chemiluminescence substrate (Pierce) and exposed to BioMax MR film (Kodak). The purified protein samples from Ni-NTA agarose and avidin affinity chromatography were concentrated, denatured and separated in a 12% SDS-PAGE gel (Bio-rad) to calculate the molecular weight of α and β chain by comparison its migrated rate to that of protein standard ladder. Also, the native purified samples were run in 12% PAGE (Bio-rad) under non-reduction conditions and stained by Imperial protein stain (Pierce) to determine whether the recombinant *Mamu-DRα* and *β* molecules could assembly of heterodimer by observing its diffuse bands.

### Purification of biotinylated Mamu-DR αβ monomer

The selected monoclonal cell line was adapted into serum-free Express Five medium (Invitrogen) supplemented with 20 mM L-glutamine and 10 µg/ml of *Blasticidin*. The expanded cells were transferred to 1 L conical flasks (Wheaton) for large-scale culture at 28°C with 120∼140 rpm in a rotary shaker, and induced to secrete expression with 500 µM CuSO_4_ for 3∼4 days when cell densities exceeded 2×10^7^/ml. The clarified supernatants were collected by centrifugation, removed free Cu^+2^ by PBS dialysis, and then concentrated by ultrafiltration with 30 kDa molecular weight cut-off Vivacell concentrator (Vivasciences). The *Mamu-DR/*P65 molecules were purified via two-round procedures as we recently described [22]. First the concentrated samples were passed through a Ni-NTA agarose affinity column (Qiagen) under native conditions and then collected the 6×His-tagged recombinant proteins by 250 mM imidazole elution. After exchange with 10 mM Tris buffer (pH 8.0), the purified samples were concentrated to 2.0 mg/mL and then biotinylated using d-biotin and *Bir*A enzyme (Avidity) in the optimal conditions. The excess biotin was removed by overnight PBS dialysis, the biotinylated molecules were purified by passing through an avidin affinity column (Pierce) as following to the supplier's recommendations. In each purification step, small portions of sample were drawn to SDS-PAGE analysis, dot-blot assays and determine protein concentration by BCA Kit (Pierce).

### Assembling of Mamu-DR αβ tetramer

This was done as we recently described [22]. Briefly, the purified biotinylated *Mamu-DR/*P65 molecules were incubated overnight at 4°C with one-fourth of its molar amount of phycoerythrin (PE)-streptavidin (eBiosciences) to allow the formation of tetrameric complexes. After buffer exchange, the reaction mixtures were passed through a Superdex^TM^ 75 10/300GL column (GE Amersham) to separate unbinding *Mamu-DR* monomers, remove free fluorescents and purify tetrameric complexes by gel filtration in Duo-flow system (Bio-rad). The tetrameric fractions were collected and stored in 150 mM NaCl/20 mM Tris (pH 8.0) supplemented with 0.5% bovine serum albumin (BSA) in the dark at 4°C for subsequent cell staining or frozen with glycerol for longer periods of storage.

### Intracellular cytokine staining for measuring Ag-specific IFN-γ^+^ CD4 T cells

1×10^6^ of PBL from naïve, BCG-vaccinated, and *Mycobacterial*-infected monkeys was stimulated with 8 µg/ml of PPD, 10 µg/ml of Ag85B whole peptide pool or 10 µg/ml of P65 in the presence of 2 µg/ml anti-CD28 (BD Biosciences, clone CD28.2) and CD49d (BD Biosciences, clone 9F10) mAb at 37°C in 5% CO_2_ for 1 hour. PBMC stimulated with PMA (200 ng/ml, Sigma)/Ionomycin (1 µg/ml, Sigma) and un-stimulated cells with 10% FBS-RPMI 1640 medium only were served as a positive and negative control respectively. The cells were incubated for 5 hours with 1 µl of Golgi plug (Brefeldin A) at 37°C with shaking and then placed at 4°C overnight. Subsequently, the cells were washed and stained with CD3-FITC (BD Biosciences), CD4-PB (eBiosource). After washing two times with 2%FBS-PBS, 200 µl of Cytofix/cytoperm solution (Becton Dickinson) was added to permeabilize cells at 4°C in dark for 45 minutes and then intracellular stained with IFN-γ PE (BD Biosciences, clone 4S) in dark for 45 minutes. Cells were fixed with 2% formalin and analyzed by flow cytometry as we previously described [24].

### Measurement of antigen-specific CD4^+^ T-cells by tetramer direct staining

The tetramer staining was done as we recently described [22]. Briefly, PBL, splenocytes or cell suspension freshly collected from individual macaques were stained with Mamu-DR*W201/P65-PE tetramers at 2.0 µg/ml at room temperature for 20 minutes, combined with fluorescence-conjugated mAb against CD4 and CD3 or CD45 (clone MB4-6D6, Miltenyi Biotech) for 10 minutes. After 2%FBS-PBS washing, the cells were fixed and gauged for tetramer-bound epitope-specific CD4^+^ T cells by flow cytometry analysis.

### The combined tetramer staining and magnetic bead enrichment approach

The tetramer-based enrichment as previous described [25] was used to enhance the ability of the tetramer to distinguish and quantitate epitope-specific CD4^+^ T cells. Briefly, 1×10^5^–1×10^7^ cells isolated from blood, spleen and periaortic lymph node (LN) of macaques were incubated with 2.0 µg/ml of Mamu-DR*W201/P65-PE tetramer at room temperature for 20 minutes. Cells were then washed with ice-cold PBS buffer. The tetramer-stained cells were then incubated with 0.1 ml of anti-PE Ab-conjugated magnetic microbeads (Miltenyi Biotech) on ice for 20 min, followed by two washes with PBS buffer. The cell suspensions were passed over a magnetized LS column (Miltenyi Biotech) and washed in the column three times with PBS buffer. The tetramer-unbound cells (washes-out) were collected to stain for CD4 and CD3 or CD45 markers for flow cytometric analysis. Then the column was removed from the magnetic field, and the tetramer-bound cells were eluted by pushing 5 ml of PBS buffer through the column with a plunger. The enriched fractions of cells were resuspended, stained for CD3 and CD4 markers, and then counted for the total number of tetramer-bound CD4 T cells in flow cytometry analysis.

### P65 stimulation and 5, 6-carboxyfluorescein diacetate succinimidyl ester (CFSE)-based proliferation

To increase sensitivity of tetramer staining of P65-specific CD4 T cells, 1×10^6^ of PBL, splenocytes or LN cell suspension was stimulated with 10 µg/ml of P65 in 10% FBS-RPMI 1640 medium at 37°C in 5% CO_2_. Cells stimulated with a SIV Gag peptide and un-stimulated cells with medium only were served as negative controls, respectively. 48 hours later, the cells were fed every 2 days with IL-2 (5 U/ml) + P65 or control peptide (10 µg/ml) until 7 or 12 days, and the cells were then harvested and incubated with Mamu-DR*W201/P65-PE tetramers at 2.0 µg/ml for staining 20 minutes. Subsequently, cells were stained with CD4, CD3 or CD45 antibodies for 10 minutes and then gated for the tetramer-binding CD4^+^ T cells as described above.

To demonstrate tetramer-specific binding to P65-specific CD4 T cells, PBL labeled with CSFE were stimulated with P65 or control peptides, and then assessed for the ability of the proliferating cells to be stained by the tetramer. Briefly, 2×10^5^/well of PBL from the MTB-infected monkeys was labeled with 2 mM/ml of CFSE (5, 6-carboxyfluorescein diacetate succinimidyl ester) using CellTrace CFSE cell proliferation kit (Invitrogen Molecular Probes) as we recently described [26]. The labeled cells were then stimulated with 10 µg/ml of P65 or control peptides at 37°C for 7 or 12 days as described above. Anti-CD3 plus anti-CD28, and medium alone were used as positive and negative controls, respectively. At end of assays, the cells ere harvested and stained by the tetramer, and CD3 and CD4 antibodies, and then analyzed by flow cytometer as recently described [26].

## Results

### Ag85B peptide 65 (P65) induced apparent proliferation in PBL during BCG infection of Mamu-DRB*W201^+^ rhesus macaques

**Figure 1:**
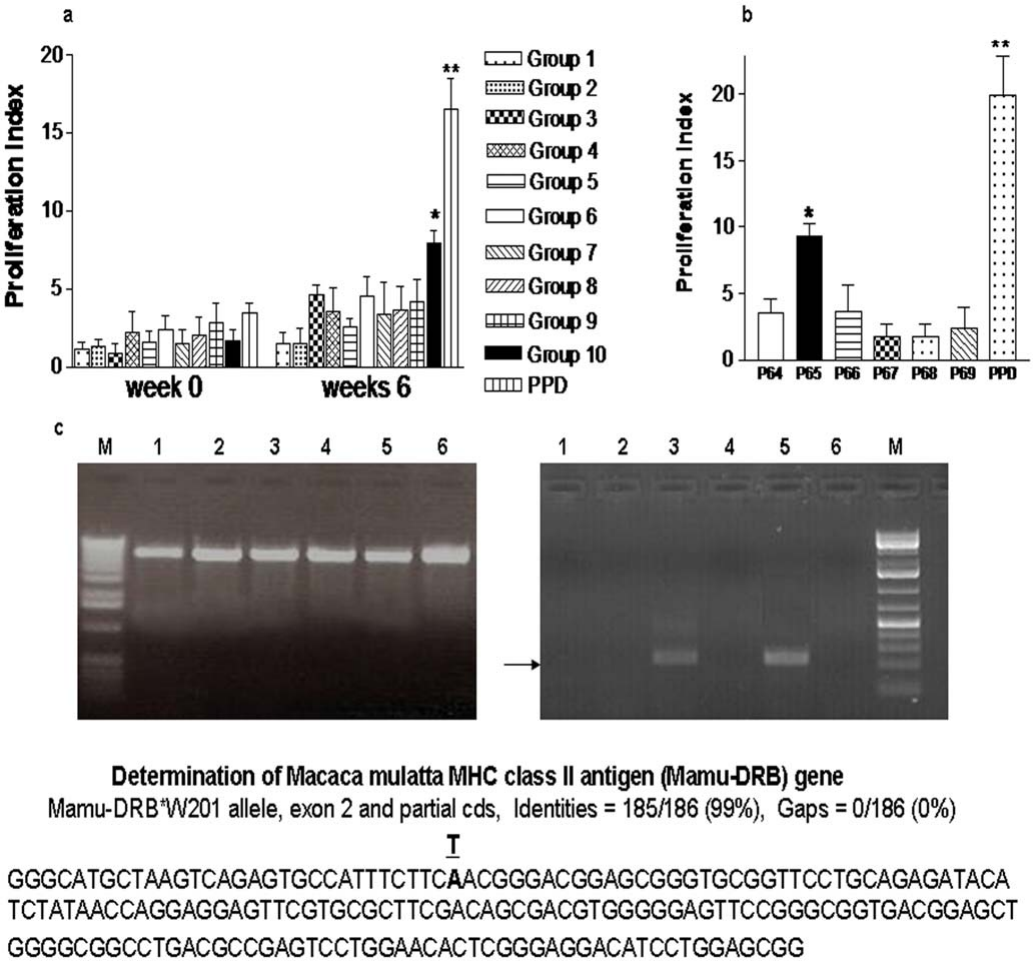
Ag85B peptide 65 induced apparent proliferation in PBL from BCG-vaccinated Mamu-DRB*W201^+^ macaques. (a) Sixty nine peptides spanning entire Ag85B protein were divided as 10 groups to examine PBL proliferation in the BCG-vaccinated macaques. The proliferation index data indicate that Ag85B Group 10 peptide pool comprised 6 overlapping peptides induced significant PBL proliferation than other peptide groups (*, p<0.05). Data were mean values derived from PBL of four BCG-vaccinated macaques, with error bars indicating standard errors of means (SEM). (b) PBL from four BCG-vaccinated macaques were further tested for their proliferation to individual peptides in the Group 10 peptide pool, and the proliferation index data reveal that PBL had stronger proliferation to the peptide #65 (P65) than other peptides (**,p<0.01; *, p<0.05). P65 bears the sequence of PNGTHSWEYWGAQLN that corresponds to 258∼272 amino acid of Ag85B. (c) Nest-PCR were used to amplify full-length *Mamu-DRB* cDNA (left gel) and, subsequently, the exon 2 (right gel) of β1 domain in DRB*W201. As illustrated, each lane represents a sample from one animal; ∼250 bp DNA fragments from the lanes 3 and 5 were excised for direct sequencing. The Mamu-DRB*W201^+^ allele was determined by sequencing alignments through Blast research of GeneBank data base. Representative DNA sequence shows that the nucleotide sequences from a rhesus macaques were identical to the Mamu-DRB*W201 prototype sequence except for one base substitution (T→A).

As an initial effort to develop tetrameric MHC/peptide complex, PBL from individual BCG-vaccinated macaques were assessed for the ability to proliferate in vitro in response to pooled 15mer peptides overlapping by 11mer and spanning entire Ag85B protein. We found that PBL from four BCG-vaccinated macaques proliferated apparently in response to Group 10 peptide pool comprised 6 overlapping peptides when compared to other peptide groups (Fig. 1a). The further individual peptide experiments allowed us to confirm that the peptide #65 (P65) bearing PNGTHSWEYWGAQLN sequence stimulated potent proliferation of PBL from the BCG-vaccinated macaques (Fig. 1b). To examine the potential P65-associated class II MHC allele shared by the four BCG-vaccinated macaques, cDNA from PBL of the macaques were detected by nest-PCR as previously described [15], [27] and sequenced the exon 2 gene encoding β1 domain of Mamu-DRB allele (Fig. 1c, 1d). Using this approach, we identified that the class II MHC gene encoding Mamu-DRB*W201 allele as designated in the nonhuman primate data base was shared by the four BCG-vaccinated macaques that developed proliferative responses to P65. All the macaques shared a single *Mamu-DRA* allele (DRA 101). These results allowed us to exploit class II MHC molecule and P65 peptide for development of Mamu-DR*W201/P65 tetramers.

### Production and characterization of Mamu-DR*W201/P65 tetramers

**Figure 2:**
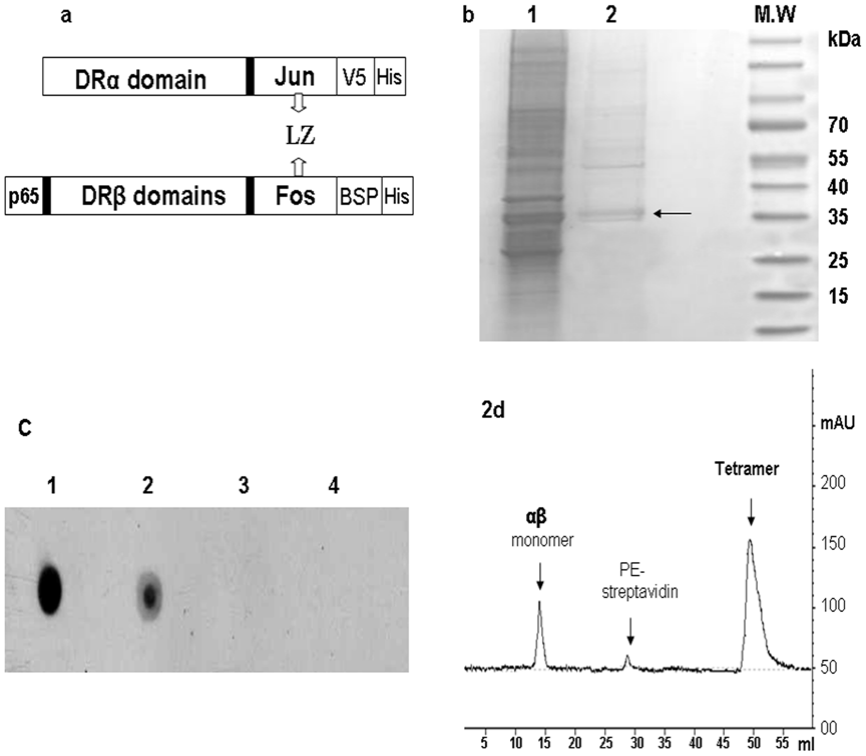
Production and characterization of soluble Mamu-DR αβ monomer and Mamu-DR*W201/P65 tetramer. (a) Schematic presentation (cartoon) of the covalent peptide approach for developing class II MHC/peptide complex constructs. The epitope-coding sequence is linked to the 5′ *Mamu-DRβ* cDNA; *Jun* and Fos-BSP are introduced by linking the extracellular domains of DR-α and -β chain, respectively. The recombinant Mamu-DR αβ monomer is stabilized by leucine zipper (LZ) formed through Jun-Fos interaction. (b) Protein samples after 1^st^ round (lane 1) and second round (lane 2) purifications were separated in the SDS-PAGE gel in reduction conditions and stained. Lane 1, the (His)_6_ tagged recombinant protein purified from Ni-NTA agarose; lane 2, the biotinylated Mamu-DR recombinants purified further through an avidin column after biotinylation. The arrow indicates two closely-separated protein bands in lane 2 (∼34, 36 kD) that correspond to predicted molecular weights of Mamu-DR α and β recombinants, respectively. (c) Dot blot assay indicates that anti-HLA-DR antibody (L243) bound to the soluble recombinant Mamu-DR αβ monomer purified by Ni-NTA affinity column (loading 1) and further by an avidin column (loading 2), but not to the denatured Mamu-DR αβ sample (loadings 3). The supernatant of non-transfected S2 cells served as a negative control (loading 4). (d) FPLC graph shows that the unbound Mamu-DR αβ molecules and free fluorescents were washed out, and the assembled Mamu-DR*W201-P65 tetramer was collected and marked as tetramer.

To produce soluble Mamu-DR αβ monomer, truncated cDNA encoding the extracellular domains of Mamu-DRB*W201 and Mamu-DRA were constructed using the PCR-based subcloning as previously described [15], [22]. To facilitate the formation of recombinant Mamu-DR αβ monomer, Jun and Fos dimerization motifs were introduced, as we recently described [22], at the 3′-end of α and β chains, respectively (Fig. 2a). To ensure that the epitope PNGTHSWEYWGAQLN was covalently attached to N-terminal of Mamu-DRβ chain for easy access to peptide-binding site, the P65 peptide-coding sequence was linked to the 5′ Mamu-DRβ cDNA (Fig. 2a). Furthermore, a short DNA fragment encoding biotinylated domain (BSP) was introduced in frame at the 3′ of the P65-β-Fos cassette for biotinylation of Mamu-DR molecules to assemble tetramer (Fig. 2a).

Soluble Mamu-DR αβ monomer secreted in supernatant from cloned S2 cells was initially purified by the Ni-NTA agarose, and then biotinylated enzymatically with the BirA enzyme at its optimal conditions. The efficiency of biotinylation was shown to be about 85%. The biotinylated protein was further purified by passing through an avidin affinity column. The two-round purification gave rise to ∼70% purity of Mamu-DR αβ monomer (Fig. 2b). To determine whether the soluble Mamu-DR αβ monomer could be recognized by anti-MHC class II mAb that reacts with HLA DR molecules on cell-surface, a dot-blot assay was carried out under the non-denatured condition. The dot blot results showed that the soluble Mamu-DR protein was clearly recognized by anti-HLA DR mAb (Fig. 2c). To assemble Mamu-DR*W201/P65-PE tetramers, biotinylated soluble Mamu-DR αβ monomers were mixed with phycoerythrin (PE)-labeled streptavidin at a molar ratio of 4∶1. Unbound monomers and PE-streptavidin were removed by gel filtration as we previously described [22]. The assembled Mamu-DR*W201/P65 tetramers were consistent with what was predicted in FPLC analysis (Fig. 2d). These purified PE-labeled tetramers were then used as staining reagent to analyze class II MHC-restricted epitope-specific CD4^+^ T cells in different samples.

### Mamu-DR*W201/P65 tetramer was able to specifically stain epitope-specific CD4 T cells in *M. tuberculosis*-infected Mamu-DRB*W201^+^ macaques

**Figure 3:**
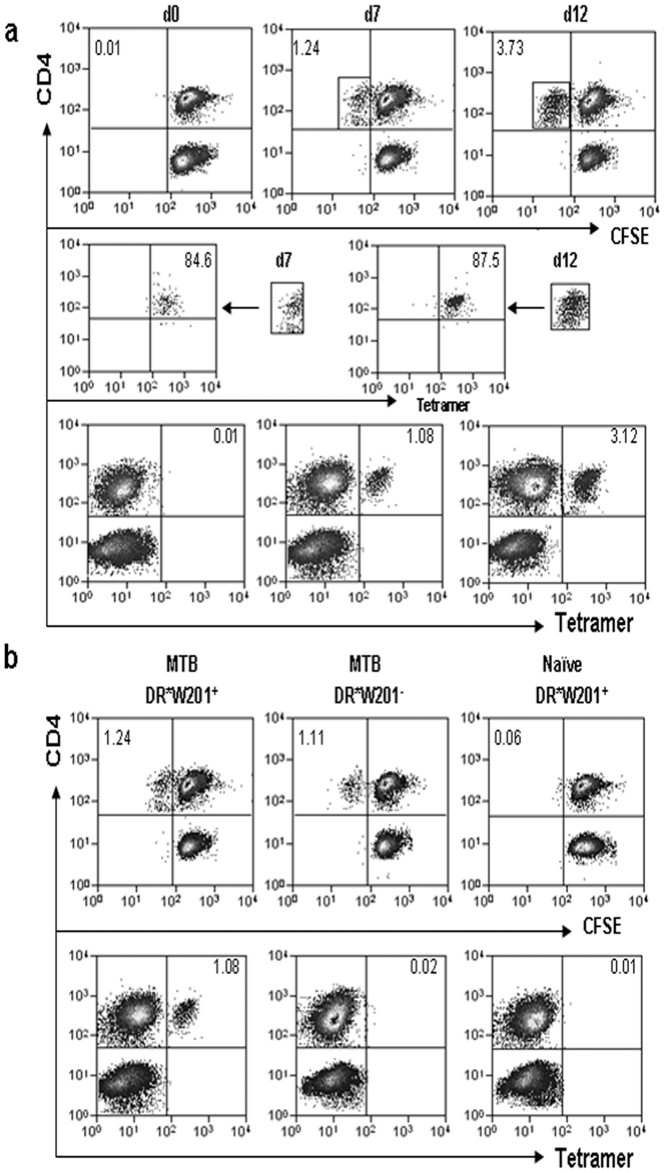
Tetramer was able to specifically stain P65-specific CD4 T cells in *M. tuberculosis*-infected Mamu-DRB*W201^+^ macaques. (a) PBL collected at day 42 from the *M. tuberculosis*-infected Mamu-DRB*W201^+^ macaque was stimulated with P65 for different days in CFSE-incorporated culture; the proliferating cells were assessed for the ability to be stained by Mamu-DR*W201/P65 tetramer. The upper panel shows CD3-gated flow cytometry histograms indicating proliferating and non-proliferating CD4 T cells as determined by CFSE dilution, with the numbers illustrated as percentages of P65-expanded CD4 T cells. The middle panel histograms indicate that majority of P65-proliferating CD4 cells as gated on CD4 and CFSE could be stained by Mamu-DR*W201/P65 tetramer. The lower panel shows CD3-gated flow histograms indicating the percentages of the tetramer-bound CD4^+^ T cells in the cultures stimulated with for 7 and 12 days, respectively. The PBL not stimulated with P65 stimulation was denoted as day 0. (b) The CD3-gated flow cytometric data show that Mamu-DR*W201/P65 tetramer specifically stains P65-proliferating CD4 T cells from the *M. tuberculosis*-infected Mamu-DRB*W201^+^ macaque but not the naïve macaque or the *M. tuberculosis*-infected Mamu-DRB*W201 ^-^ animal (lower panel).

The PE-labeled Mamu-DR*W201/P65 tetramer was then used as staining reagent to visualize P65 epitope-specific CD4 T cell responses during mycobacterial infections of rhesus macaques. To this end, we infected three naïve Mamu-DRB*W201^+^ macaques with *M. tuberculosis* to establish primary infection, and six Mamu-DRB*W201 ^−^ macaques were included as controls. PBL from *M. tuberculosis*-infected Mamu-DRB*W201 ^+^ macaques were stimulated with P65 in CFSE-based proliferation culture as we previously described [26]. The proliferating cells were then assessed for the ability to be stained by the PE-labeled tetramer. As measured by the dilution of CFSE incorporation, proliferating CD4^+^ T cells in PBL of *M. tuberculosis*-infected Mamu-DRB*W201^+^ macaques increased from ≤0.01 before stimulation to 2.07±0.54% and 6.32±0.91% after in vitro P65 stimulation for 7 and 12 days, respectively (upper panel in Fig. 3a). Interestingly, ∼85% of these proliferating CD4 T cells as gated by CD4 and CFSE were stained by PE-labeled Mamu-DR*W201/P65 tetramer (middle and lower panels in Fig. 3a). Importantly, this PE-labeled tetramer specifically bound to P65-stimulated proliferating CD4 T cells in PBL from the *M. tuberculosis*-infected Mamu-DRB*W201^+^ (positive) macaques, but not those Mamu-DRB*W201^−^ (negative) animals (Fig. 3b). The control Mamu-DR*W309/Hsp P1 tetramer was not able to stain P65-stimulated proliferating CD4 T cells in *M. tuberculosis*-infected or BCG-vaccinated Mamu-DRB*W201^+^ macaques (data not shown). These results therefore demonstrated that our generated tetramer was able to specifically stain Ag85B P65-specific CD4 T cells in *M. tuberculosis*-infected Mamu-DRB*W201^+^ macaques.

### Standard tetramer staining measured P65 epitope-specific CD4 T cells after *M. tuberculosis* infection and its proliferation after P65 stimulation

**Figure 4:**
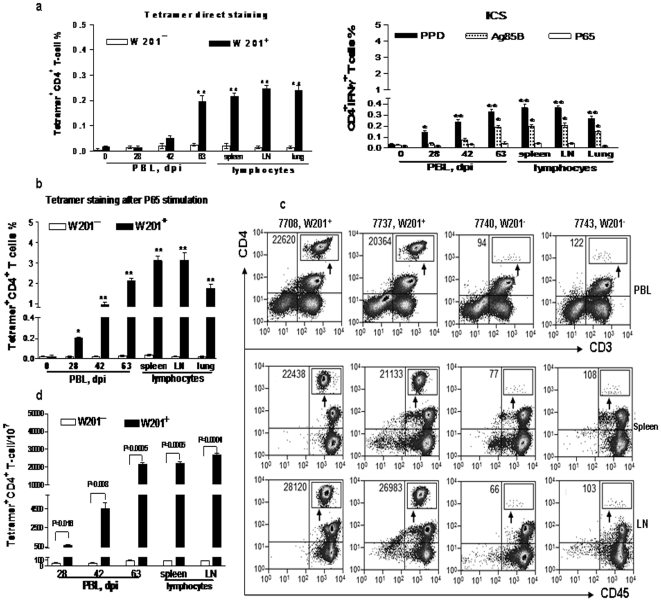
Tetramer-based enrichment conferred high-fidelity to measure P65-specific CD4 cells in comparisons of standard tetramer staining. (a) Left bar graph shows the frequencies of Mamu-DR*W201/P65 tetramer-bound epitope-specific CD4 T cells detected by standard tetramer staining in the different samples after *M. tuberculosis* infection. Standard tetramer staining detected significant increases in percentages of the P65-specific CD4 T cells in the blood, lymph nodes, spleens, and lungs compared to base line levels in blood or to those of the infected Mamu-DRB*W201 ^-^ macaques at day 63 after the infection (**, p<0.01), but no significance at days 28 and 42 post-infection. Right bar graph shows that ICS does not detect significantly-increased numbers of P65-specific IFNγ-producing CD4 T cells after *M. tuberculosis* infection. (b) The bar graph shows that the tetramer staining after P65 stimulation detects about 10-fold greater numbers of DR*W201 tetramer-bound CD4 T cells in PBL or tissue lymphocytes compared to standard tetramer staining without P65 stimulation (**,p<0.01; *, p<0.05). (c) Flow cytometry histograms show that the tetramer-based enrichment approach confers the enhanced ability to enumerate P65-specific CD4 T cells and to distinguish from background staining in 1×10^7^ PBL or tissue lymphocytes from individual *M. tuberculosis*-infected monkeys. Tetramer-unbound cells washing out from the microbead column were shown in CD4 versus CD3 or CD45 events in the flow cytometry analysis. The bead-enriched tetramer-bound cells were counted and displayed in the contour plot in the upper right CD4 quadruple, with the total numbers shown in the upper left CD4 quadruple. (d) Bar graph shows absolute numbers of Mamu-DR*W201/P65 tetramer-bound epitope-specific CD4 T cells in 10^7^ total cells detected by the tetramer-based enrichment approach in different samples. This enriched approach readily detects significant increases in numbers of the tetramer-bound CD4 T cells in blood at days 28 and 42 post-infection (P = 0.016, P = 0.008 as indicated) thanks to the much lower nonspecific staining for control samples. This is in sharp contrast to the standard tetramer staining that fails to reveal significant increases in the tetramer-bound cells at these time points due to the relatively-high background staining (Fig. 4a). Also, at day 63 after the infection, the tetramer-based enrichment approach can more dramatically distinguish the tetramer-bound cells from control nonspecific cells (P values 0.0004–0.0005) than the standard tetramer staining (P values 0.008–0.005).

The development of defined epitope-specific CD4 T cells in the blood and lymphoid tissues during primary *M. tuberculosis* infection remains poorly characterized [14], [28], [29]. At least two open questions appear to remain for direct measurement of class II MHC tetramer-bound CD4 T cells in *M. tuberculosis* infection [14], [28]: (i) given low-frequency antigen-specific CD4 T cells, how can standard tetramer staining readily distinguish the tetramer-bound CD4 T cells from background staining? (ii) how high are the frequencies of tetramer-bound epitope-specific CD4 T cells in lymphoid tissues compared to blood? To answer these straight forward questions, we employed the standard direct tetramer staining to determine frequencies of DR*W201-restricted, P65-specifc CD4 T cells in the blood and lymphoid tissues during *M. tuberculosis* infection. DR*W201/P65 tetramer-bound CD4 T cells were undetectable or <0.05% in PBL collected at days 28 and 42 after pulmonary *M. tuberculosis* infection of Mamu-DRB*W201 ^+^ macaques (left bar graph, Fig. 4a). However, at day 63 after the infection, the tetramer-bound CD4 T cells increased to almost 0.2% of CD4 T cells in blood, and 0.3% in lymphocytes from spleens and lymph nodes and lungs (Fig. 4a), indicating significant increases in relative numbers of P65-specific CD4 T cells after pulmonary *M. tuberculosis* infection of the Mamu-DRB*W201^+^ monkeys (*, p<0.05, (Fig. 4a). Of note, intracellular cytokine staining assay did not detect significantly increased numbers of P65-specific IFNγ-producing CD4 T cells after *M. tuberculosis* infection of the Mamu-DRB*W201^+^ macaques (right bar graph of 4a). Importantly, the tetramer staining after the in vitro P65 stimulation of PBL or tissue lymphocytes detected about 10-fold greater numbers of DR*W201 tetramer-bound CD4 T cells in the PBL or tissue lymphocytes (Fig. 4b). In contrast, control HSP peptide stimulation for 7 days and 12 days did not induce any increases in numbers of the tetramer-bound CD4 T cells (0.02 and 0.03 respectively, data not shown), making it possible to distinguish tetramer-bound cells from background staining. Thus, these results demonstrated that while DR*W201/P65 tetramer was useful for rapid and efficient direct-measurement of P65 epitope-specific CD4 T cells in the blood and lymphoid tissues during *M. tuberculosis* infection of Mamu-DRB*W201^+^ macaques, the tetramer staining after in vitro peptide stimulation detected 10-fold greater numbers of P65-specific CD4 T cells.

### Combined tetramer staining and magnetic-bead enrichment approach conferred high-fidelity direct-measurement of P65-specific CD4 T cells in Mamu-DRB*W201^+^ macaques

Since in vitro peptide stimulation often causes phenotypic changes, the next critical question is whether an innovative application of DR*W201/P65 tetramer could confer high-fidelity direct-measurement of the low-frequency tetramer-bound CD4 T cells without peptide stimulation. To address this question, we combined tetramer staining with magnetic bead enrichment to detect P65-specific CD4^+^ T cells in the blood and lymphoid tissues of *M. tuberculosis*-infected Mamu-DRB*W201^+^ macaques. The tetramer-based enrichment technique has recently been shown to be ultrasensitive for measuring rare Ag-specific CD4 T cells [25], [30]. For the tetramer-based enrichment, 1×10^6^, 5×10^6^, or 1×10^7^ PBL from the *M. tuberculosis*-infected macaques were incubated with Mamu-DR*W201/P65 tetramer followed by anti-PE-Ab-conjugated magnetic beads, and positively-selected by a microbead column for flow cytometry analysis. The enrichment technique allowed us to clearly distinguish and quantitate DR*W201/P65 tetramer-bound CD4 T cells in PBL from Mamu-DRB*W201 ^+^ macaques (data not shown). In fact, the numbers of DR*W201/P65 tetramer-bound CD4 T cells enriched from 10^7^ PBL, splenocytes and lymph nodes were ranged from ∼20,000 to ∼30,000 at days 63 after *M. tuberculosis* infection in Mamu-DR*W201 ^+^ macaques (Fig. 4c). In contrast, extremely small numbers of the tetramer-bound CD4 T cells (≤110 per 10^7^ cells) were nonspecifically stained after the enrichment of 10^7^ PBL, splenocytes, or lymph node cells of the control *M. tuberculosis-*infected Mamu-DRB*W201 ^−^ macaques (Fig. 4c). Importantly, while at days 28 and 42 after *M. tuberculosis* infection the standard tetramer staining method failed to distinguish the tetramer-bound CD4 T cells from background stained cells (p>0.05), the tetramer-based enrichment approach conferred high-fidelity direct measurement of ∼500 and ∼4500 tetramer-bound CD4 T cells at the two time points [*p*<0.05 and <0.01 when compared to controls (Fig. 4d)]. At day 63, the tetramer-based enrichment approach more dramatically distinguished P65-specific CD4 T cells from background stained cells in blood and tissues than the standard tetramer staining (Fig. 4d). Thus, this tetramer-based enrichment approach conferred high-fidelity direct-measurement of epitope-specific CD4 T cells in the blood and lymphoid tissues during primary *M. tuberculosis* infection of Mamu-DRB*W201^+^ macaques.

### The DR*W201/P65 tetramer-based enrichment approach detected BCG-elicited resting memory P65-specific CD4 T cells at a frequency of 2-3/10,000 in PBL

**Figure 5:**
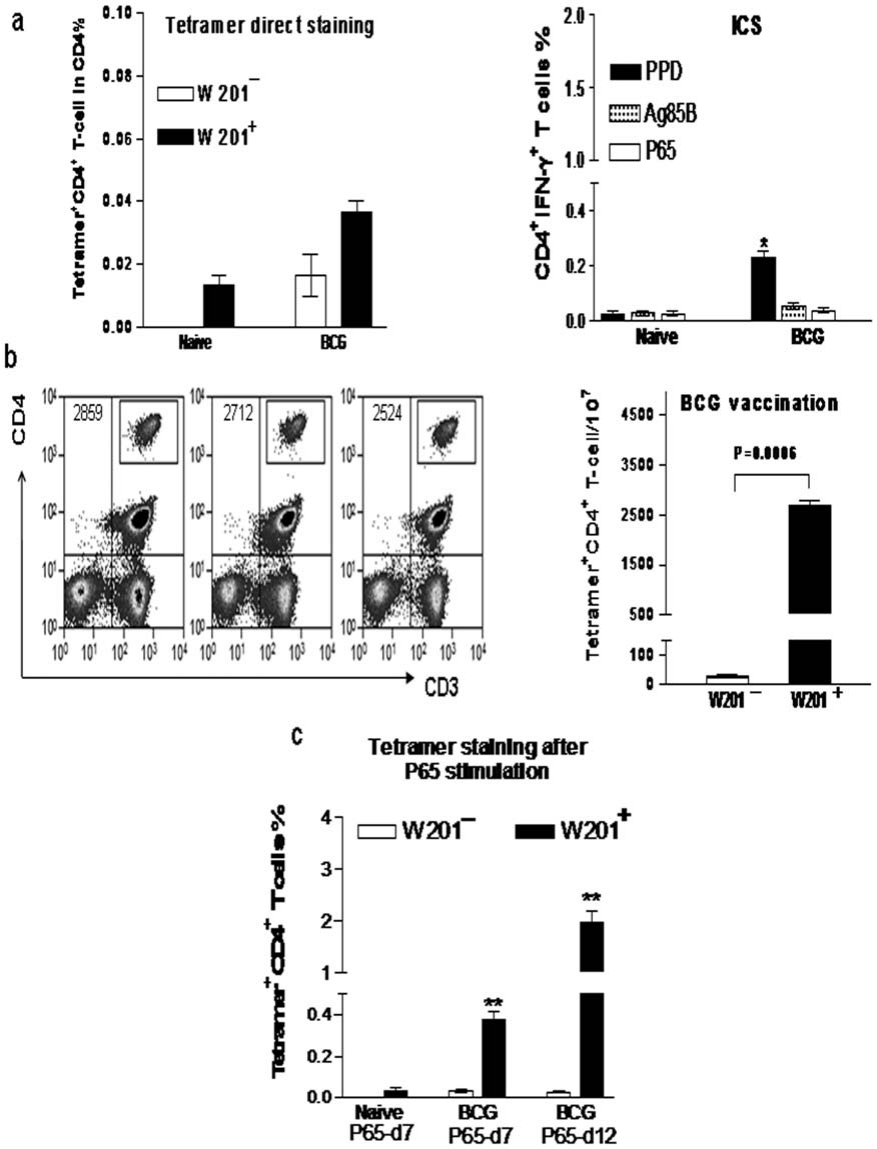
This tetramer-enriched approach detected BCG-elicited resting memory P65-specific CD4 T cells at a frequency of 2-3/10,000 in PBL. (a) Left bar graph shows that the frequency of ≤0.03% tetramer-bound CD4^+^ T-cells detected by tetramer direct staining in the BCG-vaccinated Mamu-DRB*W201^+^ macaques was difficult to distinguish from background staining (p>0.05). The bar graph on right shows that intracellular IFN-γ staining could not detect the epitope-specific IFN-γ-producing memory CD4 T cells. (b) The flow cytometry histograms on left show the total numbers of tetramer-bound epitope specific CD4 T cells detected by the tetramer-based enrichment approach in 10^7^ PBL from each macaque. The enriched tetramer^+^ CD4^+^ T-cell population is counted by flow cytometry and displayed in the contour plot in the upper right of the CD4 quadruple, with the total numbers shown in the upper left CD4 quadruple. The bar graph on right shows that this tetramer enriched approach can detect significantly greater numbers of P65-specific CD4 T cells in BCG-vaccinated Mamu-DRB*W201^+^ macaques than those in the vaccinated Mamu-DRB*W201^-^ animals. (c) The bar graph shows that the tetramer staining after P65 stimulation detects about ten-fold greater numbers of the BCG-elicited tetramer-bound CD4 T memory cells than without stimulation. The numbers of tetramer-bound cells in PBL from Mamu-DRB*W201^+^ macaques were significantly greater than those in naïve or BCG-vaccinated Mamu-DRB*W201^-^ animals (**, p<0.01).

Given the possibility that a number of new tuberculosis vaccine candidates will have to be tested and compared with BCG in macaques before moving to clinical trials, it would be necessary to determine the utility of DR*W201/P65 tetramer for precise measurement of vaccine-elicited P65-specific CD4 T cell responses. To address this straight forward question, three Mamu-DRB*W201^+^ macaques that were vaccinated for 2 years with BCG were evaluated in this study. The standard DR*W201/P65 tetramer staining method could not confidently detect vaccine-elicited P65-specific memory CD4 T cells in PBMC of the BCG-vaccinated Mamu-DRB*W201^+^ macaques, because 0.03∼0.04% frequencies were too low to distinguish them from background staining (left bar graph of 5a). Also ICS did not detect significant numbers of IFN-γ-producing CD4 T cells in the BCG-vaccinated macaques compared to the unvaccinated Mamu-DRB*W201^+^ control animals (right bar graph of 5a), despite the fact that PPD and the pooled Ag85B peptides and PPD were able to detect IFNγ-producing CD4 T cells. However, such low frequencies of DR*W201/P65 tetramer-bound CD4 T cells appeared to be truly P65-specific CD4 T cells as the tetramer-based enrichment technique showed that ∼3000 tetramer-bound cells were detected in 10^7^ PBL from the BCG-vaccinated Mamu-DRB*W201^+^ macaques, whereas <70 tetramer-bound cells were seen in controls (Fig. 5b). Similar to what was seen in *M. tuberculosis*-infected macaques, the tetramer-based flow analysis after the in vitro P65 peptide stimulation for 7 days of PBL from the BCG-vaccinated macaques detected almost 10-fold greater numbers of DR*W201/P65 tetramer-bound CD4 T cells compared to un-stimulated PBL (Fig. 5c). Thus, DR*W201/P65 tetramer staining approach was quite useful for measuring BCG vaccine-elicited memory immune responses of P65-specific CD4 T cells.

## Discussion

The current work represents a first extensive experimental study of epitope-specific CD4 T cells using class II MHC/peptide tetramer in animal models of tuberculosis. Our successful development of macaque class II MHC tetramer is indeed based on our recent production and application of Vγ2Vδ2 TCR tetramer and class I MHC tetramer [22], [27]. The Mamu-DR*W201/P65 complex appears to be adequately folded and produced by the covalent peptide approach in which the peptide is covalently linked to the β-chain for peptide placement in the peptide-binding groove during the synthesis process. Leucine zipper strategy based on Jun and Fos dimerization domains of α and β chains may also help to stabilize the folding and formation of the Mamu-DR*W201/P65 tetramer [6], [7], [9], [12]. The Mamu-DR*W201/P65 tetramer indeed made it possible to measure P65-specfic CD4 T cells directly and rapidly ex vivo during primary *M. tuberculosis* infection. It is important to note that in-depth studies of epitope-specific CD4 T cells using class II MHC tetramers for *M. tuberculosis* peptide antigens have not been reported in mice and other laboratory animals [10]. This appears to be attributed largely to the technical challenge for generating useful class II MHC tetramers. Since mouse models of tuberculosis are usually used as pioneer and proof-of-concept studies to make novel observations regarding infection and immunity, it is not surprising that current understanding of evolution and differentiation of antigen-specific CD4 T cells during *M. tuberculosis* infection of wild-type hosts is still limited. Now, the development of Mamu-DR*W201/P65 tetramers should provide a useful tool to engage many fundamental immunologic questions regarding epitope-specific CD4 T cells and their potential interrelation with Th1, Th2, Th17 and Treg differentiation during *M. tuberculosis* infection.

Both peptide stimulation and tetramer-based enrichment approaches can increase the sensitivity of DR*W201 tetramer-based measurement of P65-specific T cells. Earlier reports demonstrate that class I tetramer direct staining can pick up 0.1–1% of antigen-specific CD8 T cells in the blood, whereas in most cases class II tetramer-bound CD4 T cells are usually much lower or indistinguishable from background staining [10]. Our studies have shown that the tetramer staining after peptide stimulation can lead to an almost 10-fold increase in detection levels of Mamu-DR*W201/P65 tetramer-bound CD4 T cells in the blood compared to the standard direct staining. Importantly, we have shown that the combined tetramer-staining and magnetic-bead-enrichment approach appears to unequivocally discriminate the tetramer-bound CD4 T cells from the background-stained cells. This combined approach indeed makes it possible to directly enumerate P65-specific CD4 T cells in the blood during early time points of *M. tuberculosis* infection, circumventing the ambiguous measurement of antigen-specific T cells in acute infection. In fact, this approach is doable for using less than 10^6^ PBL (data not shown) [25], [30]. Since the tetramer-based enrichment does not require in vitro antigen stimulation or other manipulations, this approach should be useful for directly measuring dynamic changes in numbers, phenotypes and differentiation of antigen-specific CD4 T cells after infections or vaccination.

The current study demonstrates for the first time that class II MHC/peptide tetramers can confer visualization of mycobacterial epitope-specific CD4 T cells not only in the blood but also in lymphoid tissues and lungs. In fact, our data provide first evidence indicating immune distribution of single epitope-specific CD4 T cells in the blood, lymph node, spleen and infected lung after pulmonary *M. tuberculosis* infection. Interestingly, P65-specific CD4 T cells appear to be little more readily detected by the tetramer in lungs, lymph nodes and spleens than in the blood of Mamu-DRB*W201 ^+^ macaques two months after *M. tuberculosis* infection, despite no statistical significance for the difference (data not shown). Our results derived from the tetramer-based analysis are quite contrasted to what is found after *M. tuberculosis* infection of BALB/c mice, in which T cells specific for peptides spanning entire Ag85B or Ag85C protein are not detectable in spleens by intracellular cytokine staining assays [29]. Moreover, our results suggest that the lymph nodes and spleens harbor many antigen-specific CD4 T cells, and should not be ignored when counting their frequency during *M. tuberculosis* infection. Although there is limitation for a single CD4 T-cell epitope, our finding indeed raises the possibility to determine whether the DR*W201/P65-bound CD4 T cells have different phenotype, differentiation or effector function in the different anatomic compartments during pulmonary *M. tuberculosis* infection.

The development of Mamu-DR*W201/P65 tetramer should facilitate evaluation of new tuberculosis vaccine candidates in nonhuman primates. It is generally believed that resting memory CD4 T cell population are typically present at much lower frequency, beyond the detection limit by the standard tetramer staining method. In the current study, macaques vaccinated for >2 years with BCG do not exhibit P65-specific CD4 T cells that can be confidently detected by intracellular IFN-γ staining or by direct DR*W201/P65 tetramer staining. However, after P65 stimulation for 7 days or 12 days, up to 0.4 and 1.7% P65-specific CD4 T cells can be detected in PBL by the tetramer, respectively. More importantly, the combined tetramer staining and magnetic-bead enrichment approach can clearly pick up the tetramer-bound P65-specific CD4 T cells in PBL from the BCG-vaccinated macaques. These results demonstrate the utility and value of Mamu-DR*W201/P65 tetramer for precise direct-measurement of vaccine-elicited memory CD4 T cell responses years after vaccination. It is important to note that Mamu-DRB*W201 is one of several common alleles that are able to present peptides to CD4^+^ Th cells [14], [31]. This allele appears to be seen at a high frequency in Indian rhesus monkeys, accounting for 35% [15]. Our initial screening shows that ∼22% of Chinese rhesus and ∼10% of cynomolgus macaques from different colonies express the Mamu-DRB*W201 allele. On the other hand, since Ag85B has been shown to be protective antigen in macaques [32], it is useful to target and measure detectable-frequency of Ag85B P65-specific CD4 T cells in vaccine efforts. Thus, Mamu-DR*W201/P65 tetramer in combination with other assays should provide a useful system for in-depth studies of immune biology of antigen-specific CD4 T cells in the context of vaccine testing and *M. tuberculosis* infection.
